# Low Dose NeuroD1 Delivery in Mouse TBI Models

**DOI:** 10.1007/s12035-026-06244-6

**Published:** 2026-10-05

**Authors:** Isaac H. Clark, Ansharah Khan, Christopher With, Zachary Roushdy, Dilmareth Natera-Rodriguez, Kevin Sun, Olivia Erlanson, Shaimaa Khedr, Walter C. Low, Andrew W. Grande

**Affiliations:** 1 Biomedical Engineering Graduate Program, University of Minnesota, 2001 Sixth St., SE, Minneapolis, MN 55455 USA; 2 Department of Neurosurgery, University of Minnesota, 2001 Sixth St., SE, Minneapolis, MN 55455 USA; 3 Stem Cell Institute, University of Minnesota, Minneapolis, MN 55455 USA; 4 Graduate Program in Neuroscience, University of Minnesota, Minneapolis, MN 55455 USA; 5 Department of Histology, Faculty of Medicine, Ain Shams University, Cairo, 11591 Egypt

**Keywords:** Traumatic brain injury, NeuroD1, Neuronal differentiation

## Abstract

Traumatic brain injury (TBI) often results in serious neuronal tissue damage that can lead to progressive neurological impairment. NeuroD1, a transcription factor involved in neuronal differentiation, may be able to facilitate recovery post-TBI through the promotion of both neuroprotection and astrocyte-to-neuron reprogramming. The efficacy and optimal parameters for NeuroD1-based treatment, however, remain uncertain. This study investigated the effects of AAV-mediated NeuroD1 delivery in TBI mouse models, focusing on cellular reprogramming at low dosages. Dose response experiments revealed that lower viral doses provided the greatest transduction specificity. Notably, lower viral titers resulted in greater astrocyte-specific transduction whereas higher titers increased transduction in off-targets (neurons), potentially due to toxicity and/or leakage. Using these low dosages, our findings indicate that NeuroD1 treatment did not produce statistically significant structural restoration, nor did it greatly alter neuroinflammation measures; however, it did allow for functional improvement in beam walk performance, suggesting enhanced neurological recovery. Prospective studies should refine experimental conditions, incorporate a broader range of behavioral assessments, and explore combination therapies to enhance both functional and structural recovery. NeuroD1-based interventions remain a promising avenue for promoting functional improvement following TBI, although further research is required to drive these findings to clinical applications.

## Introduction

Traumatic brain injury (TBI) remains a leading cause of morbidity and mortality worldwide [1]. This has prompted various investigations into potential therapeutic options. TBI results from external mechanical injury to the head, which can lead to dysfunction in brain activity. This condition has two mechanisms as part of its pathophysiology: the primary mechanism that occurs at the time of insult and the secondary mechanism that involves a cascade of immunological processes that exacerbate tissue damage over time. Therapeutic options mostly focus on symptomatic management, rehabilitation, surgical or thrombolytic therapy, mechanical thrombectomy, and rehabilitation.

NeuroD1 is a transcription factor that plays a crucial role in the development and differentiation of neuronal cells and has been proposed as a potential treatment for neuronal tissue damage in brain injuries [2]. Studies suggest that NeuroD1 can reprogram nonneuronal cells, such as astrocytes into functional neurons. This reprogramming could offer a novel approach for repairing damaged neuronal tissues in TBI [3]. However, controversy exists regarding reprogramming capabilities and optimal therapeutic concentrations. It is unclear whether these beneficial effects are primarily due to cellular reprogramming or due to other confounding factors.

While some studies have demonstrated its efficacy in neuronal protection, others have suggested a potential for cellular reprogramming [4–6]. The controversy surrounding NeuroD1’s role in cellular reprogramming versus neuroprotection poses challenges in determining the optimal concentrations for therapeutic efficacy [7]. Balancing neuroprotection with reprogramming potential is crucial to maximizing NeuroD1 therapeutic benefits in TBI [8]. This study aimed to examine the existing methods of NeuroD1 delivery in TBI models, explore the effect of virus dose on transduction specificity, deduce the potency of each variation, and where future directives can proceed.

## Methods

### Animals

All animals in this project were C57BL/6 mice. Animals in the alternate dose transduction experiment were male aged 2–4 months. Animals in the astrocyte-to-neuron reprogramming with low dose in the TBI model experiment were evenly split male/female and aged 2–4 months (Table 1).

**Table 1:** Sample sizes and sex of groups for experimental groups

Experimental group	Sex	Sample size
Alternate dose transduction experiment		
Control virus injection (10^11^ GC/mL)	Male	3
Control virus injection (10^12^ GC/mL)	Male	3
Control virus injection (10^13^ GC/mL)	Male	3
Astrocyte-to-neuron reprogramming with low dose in TBI models		
TBI + control virus	Male	7
TBI + control virus	Female	6
TBI + treatment virus	Male	7
TBI + treatment virus	Female	7

### Viruses

#### AAV9-GFAP(short)-Cre: 2.4E13 GC/mL + AAV9-CAG-DIO-NeuroD1-T2A-mRuby2: 2.5E13 GC/mL

**Fig. 1:**
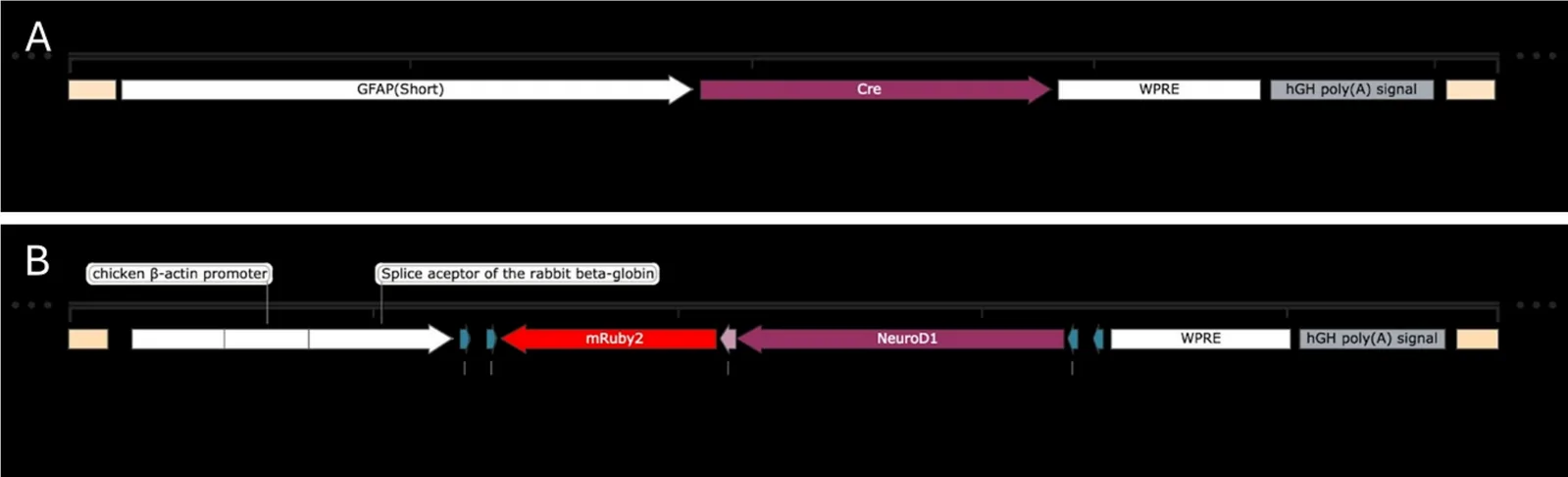
Transgenes for viral system. **A** AAV9-GFAP(short)-Cre. **B** AAV9-CAG-DIO-NeuroD1-T2A-mRuby2

These constructs were designed for the purpose of inducing cellular reprogramming in endogenous astrocytes (Fig. 1). These were mixed evenly prior to injection. Glial fibrillary acidic protein promoter (GFAP) is a promoter sequence that drives GFAP expression. The GFAP protein is found commonly in astrocytes over other cell types, and as such, the GFAP promoter is more active in astrocytes. Cre recombinase (Cre) is a bacteria-derived, type I topoisomerase that can be used with LoxP to make site-based genetic switches [9]. Cre recombinase can be used for genetic modifications such as excising DNA segments or flipping sequences by mediating site-specific recombination between LoxP sites. To prevent unintended transcription, the genes of interest in the second vector were inverted within double-floxed inverted open reading frames (DIO). Cre recombinase would then flip the sequences into a readable orientation and subsequently remove the LoxP sites, locking the modification in place permanently. The CAG promoter (CAG) was selected due to its capabilities in driving high amounts of expression in all mammalian cells [10]. CAG paired with the genetic switch ensures that the genes of interest will continue to be expressed, regardless of the cell type and resulting regulation of the GFAP promoter. Without this switch, a cell identity change could reduce activity of the GFAP promoter, halting NeuroD1 production and potentially leading to partial reprogramming. NeuroD1 was included due to its potential in converting responsive glial cells into viable neurons in mouse brains [11]. Finally, mRuby2 was added to this vector to allow its tracking and the identification of transferred cells.

#### AAV9-GFAP(short)-Cre: 2.4E13 GC/mL + AAV9-CAG-DIO-mRuby2: 1.0E13 GC/mL

**Fig. 2:**
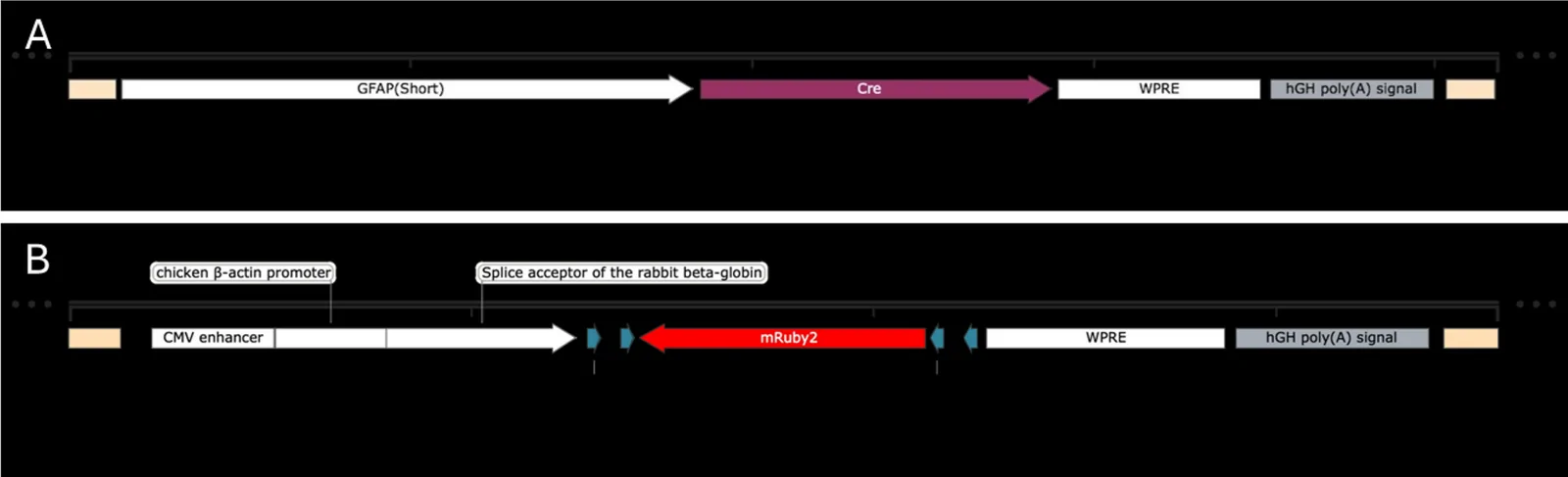
Transgenes for viral system. **A** AAV9-GFAP(short)-Cre. **B** AAV9-CAG-DIO-mRuby2

These constructs were designed for the purpose of marking endogenous astrocytes in the same manner as the sequences designed for reprogramming (Fig. 2). These sequences were identical to those previously described with the exception of excluding NeuroD1. Allowing for transduction of the cells and marking but no reprogramming.

### Surgeries

#### Controlled Cortical Impact

Surgeries began by prepping mice with an injection of slow-release buprenorphine (2 mg/kg). After 2 h, mice were placed into an anesthesia chamber (1.5–5% isoflurane) until fully anesthetized. In the meantime, the Leica Stereotaxic Impactor Control Unit (model 39463920) was prepped by setting the velocity adjustment to 6 m/s (± 0.05), attaching the Leica Stereotaxic Impactor Actuator to the stereotaxic arm, and then checking that the “retract,” “off,” “extend,” and “impact” functions were working properly. After being anesthetized, mice were then transferred to the surgical field, where ear bars and nose cones were secured and lubricants were applied to the eyes. Nair™ hair removal and depilatory cream (active ingredients: potassium thioglycolate and calcium hydroxide) was applied to the heads of the mice for 1 min. Nair™ hair removal and depilatory cream were then wiped off with betadine and alcohol wipes. Anesthetic depth was then assessed through the pinch method. After confirming adequate anesthesia using a toe pinch reflex test, incisions were made through the use of a scalpel through the skin of the mice in order to reveal the bregma. A > 2 mm diameter craniectomy was then performed at M/L + 1.5 mm and 0.5 mm A/P. To improve TBI consistency, an electronic contact sensor attachment for the impactor was used for confirmation of the brain surface. After depth was established, TBI was generated through the controlled cortical impact with velocity 6 m/s, dwell time 100 ms, depth 1 mm, and probe diameter 2 mm. The incision was closed using sutures or staples. The ear bars and nose cones were then removed, and the mice were placed in a cage with a heating pad underneath.

#### Virus Injection

Surgical preparation was the same as other surgical procedures. The injector rate and volume were set to 0.5 µL/min and 1.5 µL, respectively. After surgical preparation, incisions were made through the use of a scalpel to the skin of the mice in order to reveal their bregma. The impactor was then utilized to measure the bregma positioned M/L ± 1.5 and A/P + 0.5. The needle was progressively lowered onto the brain’s surface, then further lowered to a depth of 2.5 mm into the brain, at which point 1.5 µL of the virus was injected. The needle was carefully raised to 1 mm, and another 1.5 µL of the virus was injected. The needle and Hamilton syringe were then carefully withdrawn. The previously incised skin was then closed using sutures or staples.

### Behavior

#### Beam Walk

The beam walk test was utilized to assess individual limb function in mice. We used 1 inch and ¼ in-thick rectangular beams with start and end markers 2 ft apart. A bright light was positioned at one end of the beam, while a box was positioned on the other end. All mice were allowed to acclimate to the room for 30 min prior to the beginning of testing. Mice were further acclimated to the box at the beginning and end of the test for 15+ seconds. After initial acclimation, mice were placed on the far end near the bright light. Time to cross the start to end markers was recorded as well as foot faults for each animal. Foot faults were recorded as general and not specified for each foot. Each animal was given four trials per day to walk the length of the beam in 60 s or less. Training consisted of four trials each day over the course of 3 days. On the last training day, baseline results were recorded for each mouse 7 days prior to any procedures. Results were divided by the baseline to normalize all data.

#### Rotarod

The rotarod test was used to assess neurological deficits. All mice were allowed to acclimate to the room for 30 min prior to the beginning of testing. The test was performed over a period of 1 min, starting speed of 4 RPM, and acceleration of 1 RPM per second. Results were recorded as the time it takes to fall. Training consisted of four trials per day over the course of 3 days. Mice had baseline results recorded on the last day of training, 7 days prior to any procedures. Results were divided by the baseline to normalize all data.

### Tissue Processing

#### Tissue Fixation

All mice were anesthetized using isoflurane and euthanized through perfusion with PBS and 4% PFA and then decapitated. After being removed, their brains were incubated in 4% PFA for 24 h at 4 °C. The brains were incubated in 10% sucrose for 24 h at 4 °C to create a sucrose gradient. The brains were incubated in 20% sucrose for 24 h at 4 °C the next day. The brains were incubated in 30% sucrose for 24 h at 4 °C on the last day.

#### Tissue Cryosectioning

The mice brains were embedded in OCT the next day, and 20 μm-thick brain cryosections were made using a Leica CM1850 cryostat. Sections were alternated between 10 different slides. Two sets of 10 slides were used per brain, allowing us to section from ~  + 3.2 mm bregma to ~ −3.08 mm bregma. This made for a total of 20 different slides per animal.

#### Tissue Staining

Sections were dried for 10–30 min, permeabilized with PBST (0.3% Triton X-100) for 30 min, and blocked with 5% donkey serum for 45 min. After that, slices were incubated with primary antibodies against NeuroD1 (1:250 Abcam, ab205300), GFAP (1:500, Invitrogen, 13–0300), and NeuN (1:500, Abcam, ab177487), IBA1 (1:250, Wako, 011–27991) overnight at 4 °C in 5% donkey serum blocking solution. Sections were washed 3 × 5 min with PBST (0.1% Triton X-100) and then incubated with secondary antibodies labeled with Alexa Fluor 488, 555, or 647 (1:1000, Abcam) and DAPI (1:1000) in 5% donkey serum for 1 h. Sections were rinsed 3 × 5 min with PBS. Following the final rinsing, the slices were wet mounted and imaged using a Leica DMI6000 B microscope.

### Tissue Quantification

#### TBI Lesion Quantification

**Fig. 3:**
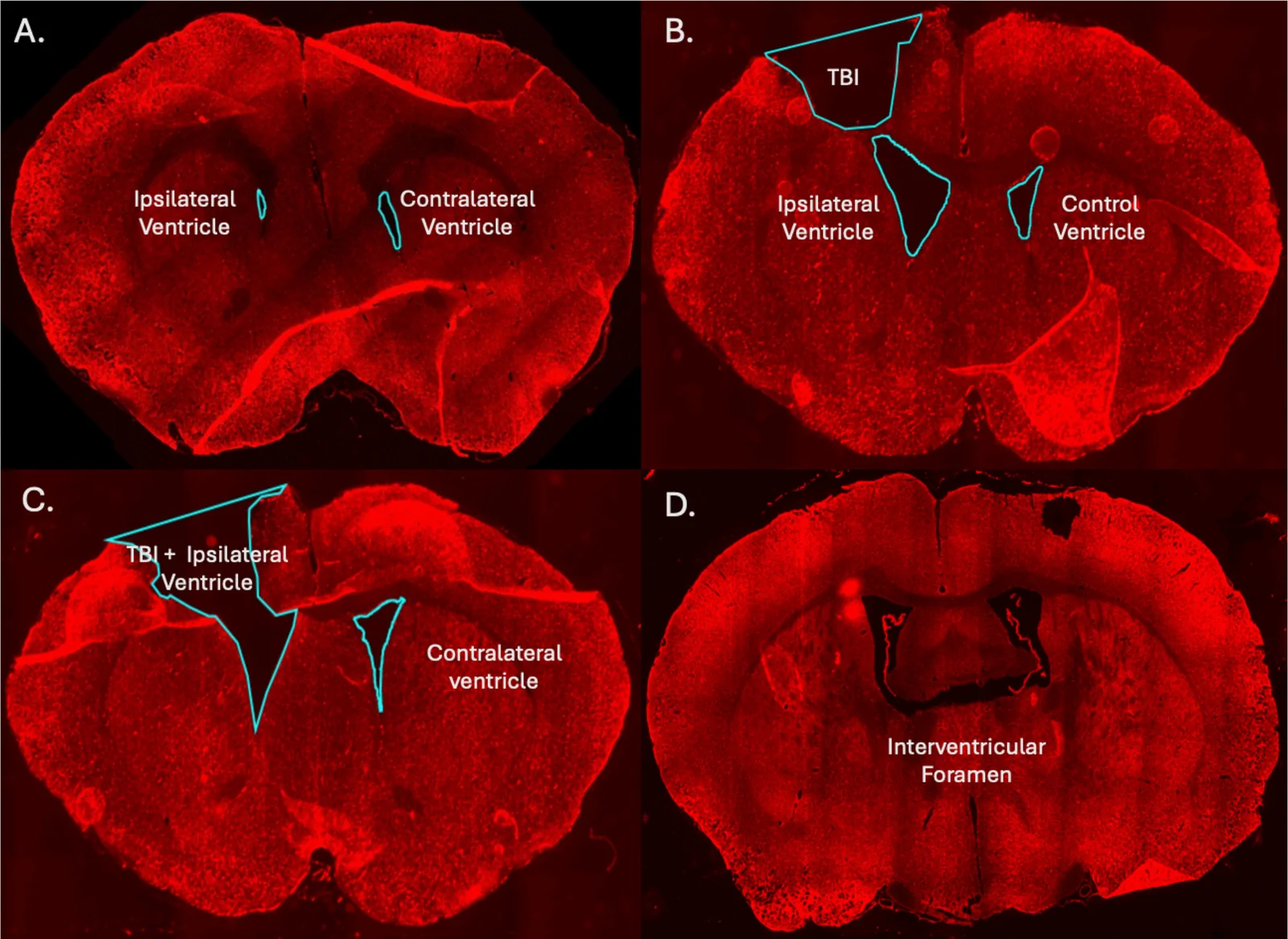
Representative brain sections illustrating ventricle and TBI lesion annotations across different anatomical regions. **A** Anterior brain section displaying intact bilateral ventricles (start point for annotations). **B** Brain section displaying ventricles and TBI with annotations. **C** Brain section displaying a ruptured ventricle with merged TBI + ventricle annotation. **D** Posterior brain section displaying lack of TBI and appearance of the interventricular foramen (end point for annotations)

To determine TBI lesion volume with QuPath, glass slide series were tile-scanned entirely using a single channel or brightfield. TBI lesions were annotated from their most anterior appearance to their most posterior appearance (Fig. 3). If a ventricular rupture occurred, an annotation was made to represent the combined area of the TBI and ventricle. The areas of those annotations were then multiplied by the section thickness and number of slide series to find the total volume.

#### Ventricle Volume Quantification

To determine ventricle volume, the tile-scanned images previously acquired were used again. Ventricles were annotated from their first most anterior appearance until the interventricular foramen became visible. If a ventricular rupture occurred, the combined TBI and ventricle annotation was used for that slice’s calculation. The areas of those annotations were similarly multiplied by the section thickness and number of slide series to find the total volume.

#### Cell Quantification

To determine co-labeling and cell morphology, QuPath software was used for analysis of the stained tissue sections [12]. Whole coronal slice microscopy tile scans were acquired near the TBI site and annotation areas were made either around the entire tissue section (in the case of glial cell quantification) or specific regions of interest (in the case of mRuby2 quantification). mRuby2 expression tended to be localized predominantly located within layers V/VI of the primary motor cortex, with additional labeling observed in the corpus callosum and primary somatosensory cortex. Region of interest annotations were manually drawn around any and all clear cases of mRuby2 (typically areas < 500 µm^2^) within the cryosectioned range. For cell detection, adjustments for cell size and intensity were made as needed on the highest quality image for each mouse. Single measurement classifiers were used to identify colabeled cells, in which an appropriate threshold was chosen to identify the majority of colabeled cells. After all classifiers were made, they were applied to allow for the identification of all channel co-labeling. To ensure accuracy, researchers manually adjusted cell identification/classification parameters based on visual inspection, until the majority of QuPath identified cells were correctly identified/classified. This process did not eliminate all misclassifications but rather provided a best estimate representation of cell populations. For cell morphology, nonreactive glial cells were determined as those lacking clear signs of hypertrophy and being most similar to those found in a noninjured sample. Nonreactive cells were most often found on the side contralateral to any injury and on the inferior portion of the brain (near the nucleus accumbens and substantia innominata). This brain region was used to determine the average morphology of a nonreactive cell in each sample. The average soma size and standard deviation was used to calculate an approximate threshold for morphological reactivity, such that anything above that threshold would be statistically unlikely to be in the same group as nonreactive glial cells. This threshold was defined as two standard deviations above the mean soma area (threshold = average + 2 × standard deviation).

### Statistics

In all cases, comparing animals treated with NeuroD1 to controls, single-tailed *t* tests were used. In cases comparing differences between alternate dose co-labeling, two-factor ANOVA with replication was used across all groups, and additional post hoc multiple-comparison testing using Sidak correction was performed. Error bars in figures represent standard error.

## Results

### Alternate Dose Transduction

#### Experimental Design

**Fig. 4:**
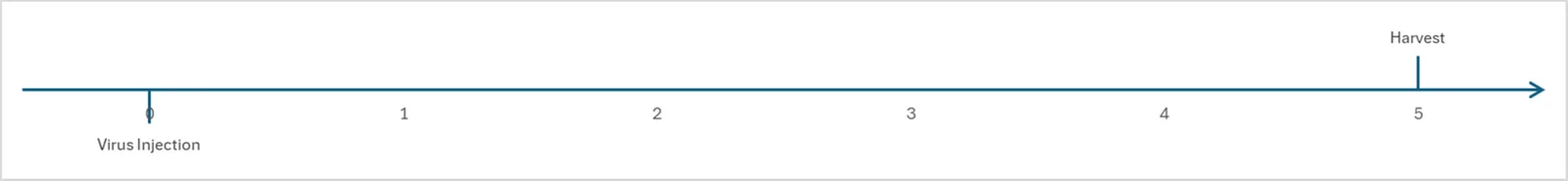
Timeline for alternate dose transduction experiment

To find the optimal virus dosage, we began by conducting an experiment in which the dosage of AAV was varied. The viral dose can greatly affect the transduction efficacy [13], and in particular, it has been seen that high doses of AAV have toxic side effects resulting in cell death and apparent leakage of the expressed proteins. Lower doses, however, have shown greater transduction specificity rates compared to higher doses. To validate this, we sought to explore how different doses of our virus would affect which cells expressed our tracking biomarker mRuby2. We injected our control viral system AAV9-GFAP(short)-Cre + AAV9-CAG-DIO-mRuby2, varied the dose from 10^11^, 10^12^, and 10^13^ GC/mL, and harvested all groups 5 days later (Fig. 4). With this system, the expression should be limited to astrocyte cells that naturally have the GFAP promoter highly active.

#### co-labeling

**Fig. 5:**
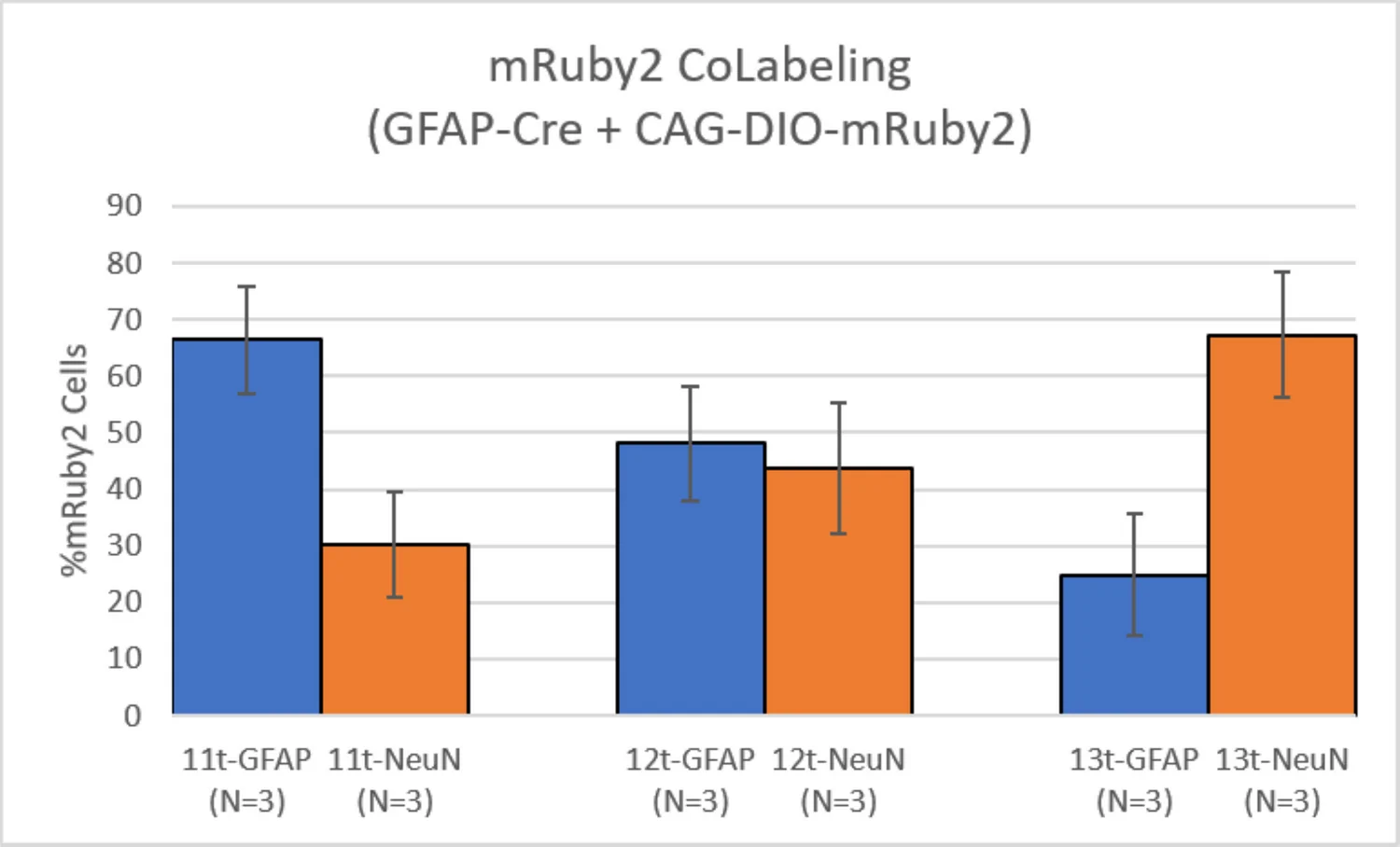
mRuby2 co-labeling with control virus at alternate doses

To analyze which cells expressed mRuby2, tissue was processed and stained for GFAP and NeuN. A two-way ANOVA was performed to examine the effects of dose on mRuby2 co-labeling with GFAP and NeuN. A significant interaction between viral dose and co-labeling was observed (*F*(2,12) = 4.74279, *p* = 0.03035), indicating that the increase in viral dose differentially affected astrocyte and neuronal transduction. Consistent with previous reports, lower doses resulted in greater astrocyte-specific transduction, whereas increasing viral dose was associated with reduced GFAP co-labeling and increased NeuN co-labeling (Fig. 5). Post hoc multiple-comparison testing using Sidak correction, however, did not identify statistically significant pairwise differences between dose groups. These findings suggest that increasing viral dose decreased astrocyte-specific transduction while increasing off-target neuronal transduction, supporting the use of lower viral doses to maximize transduction specificity, although individual dose comparisons do not show statistical significance after correction.

### Astrocyte to Neuron Reprogramming with Low Dose in TBI Models

#### Experimental Design

**Fig. 6:**
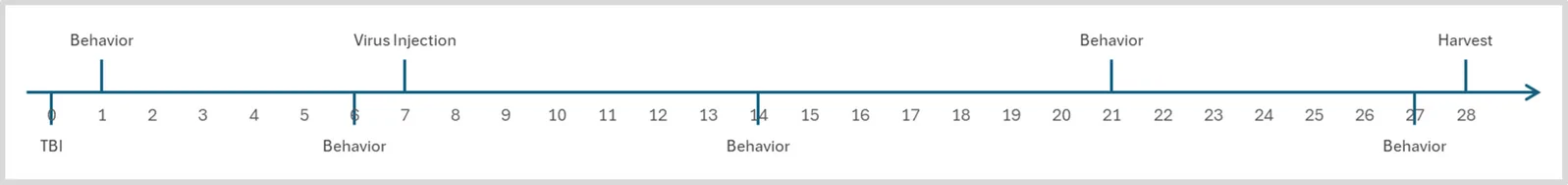
Timeline for astrocyte-to-neuron reprogramming with low dose in TBI model experiment

To capitalize on the findings in the previous experiment, the effects of a low-dose NeuroD1 reprogramming treatment in TBI mice were explored. Control TBI mouse models were given two viral vectors: AAV9-GFAP(short)-Cre: 2.4^11^ GC/mL and AAV9-CAG-DIO-mRuby2: 2.5^11^ GC/mL, while treatment TBI mouse models were given AAV9-GFAP(short)-Cre: 2.4^11^ GC/mL and AAV9-CAG-DIO-NeuroD1-T2A-mRuby2: 1.0^11^ GC/mL. TBI was induced on day zero, virus injections were performed on day 7, and animals were euthanized on day 28 (Fig. 6).

#### Qualitative and Quantitative co-labeling

**Fig. 7:**
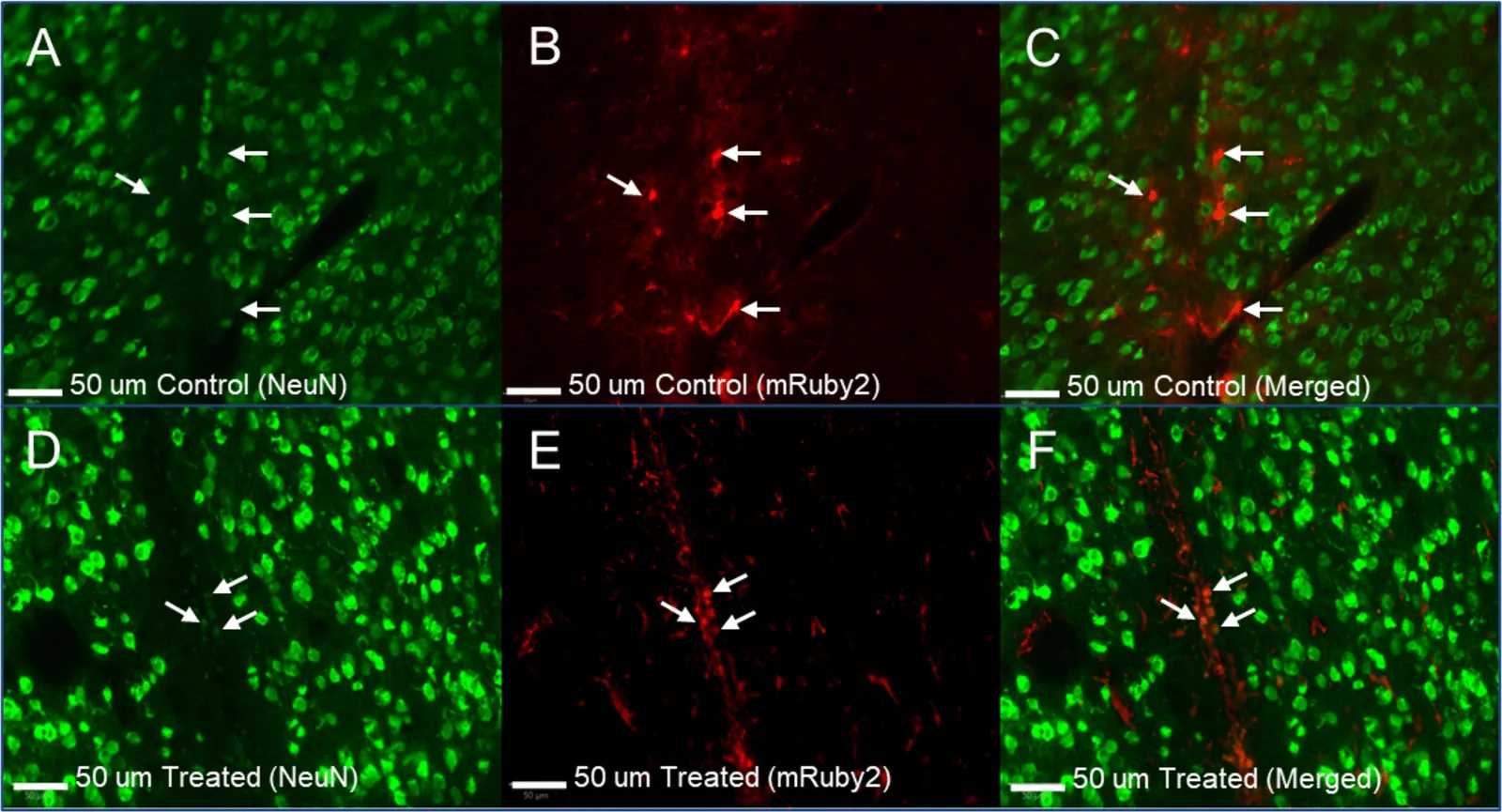
Qualitative depiction of NeuN and mRuby2 co-labeling. White arrows labeling mRuby2-positive cells. **A** Control tissue with NeuN channel displayed. **B** Control tissue with mRuby2 channel displayed. **C** Control tissue with NeuN and mRuby2 channels displayed. White arrows in **A**–**C** show mRuby2-positive cells that are NOT colabeled with NeuN. **D** Treated tissue with NeuN channel displayed. **E** Treated tissue with mRuby2 channel displayed. **F** Treated tissue with NeuN and mRuby2 channels displayed. White arrows in **D**–**F** show mRuby2-positive cells that are colabeled with NeuN

To determine if the virus was transducing cells as expected and promoting reprogramming, staining for GFAP and NeuN was conducted. It was found that in the control, evident mRuby2-positive cells did not have high levels of NeuN co-labeling, while treated samples did have NeuN co-labeling (Fig. 7A–F). Quantifying these results, it was found that the higher levels of mRuby2 and NeuN co-labeling in treated samples were statistically significant when compared with the control (Fig. 9). In contrast, mRuby2 co-labeling with GFAP was lower in the treated animals compared to the control, which was also found to be statistically significant (Fig. 9). These findings are consistent with preferential astrocyte transduction rather than direct neuronal transduction.

**Fig. 8:**
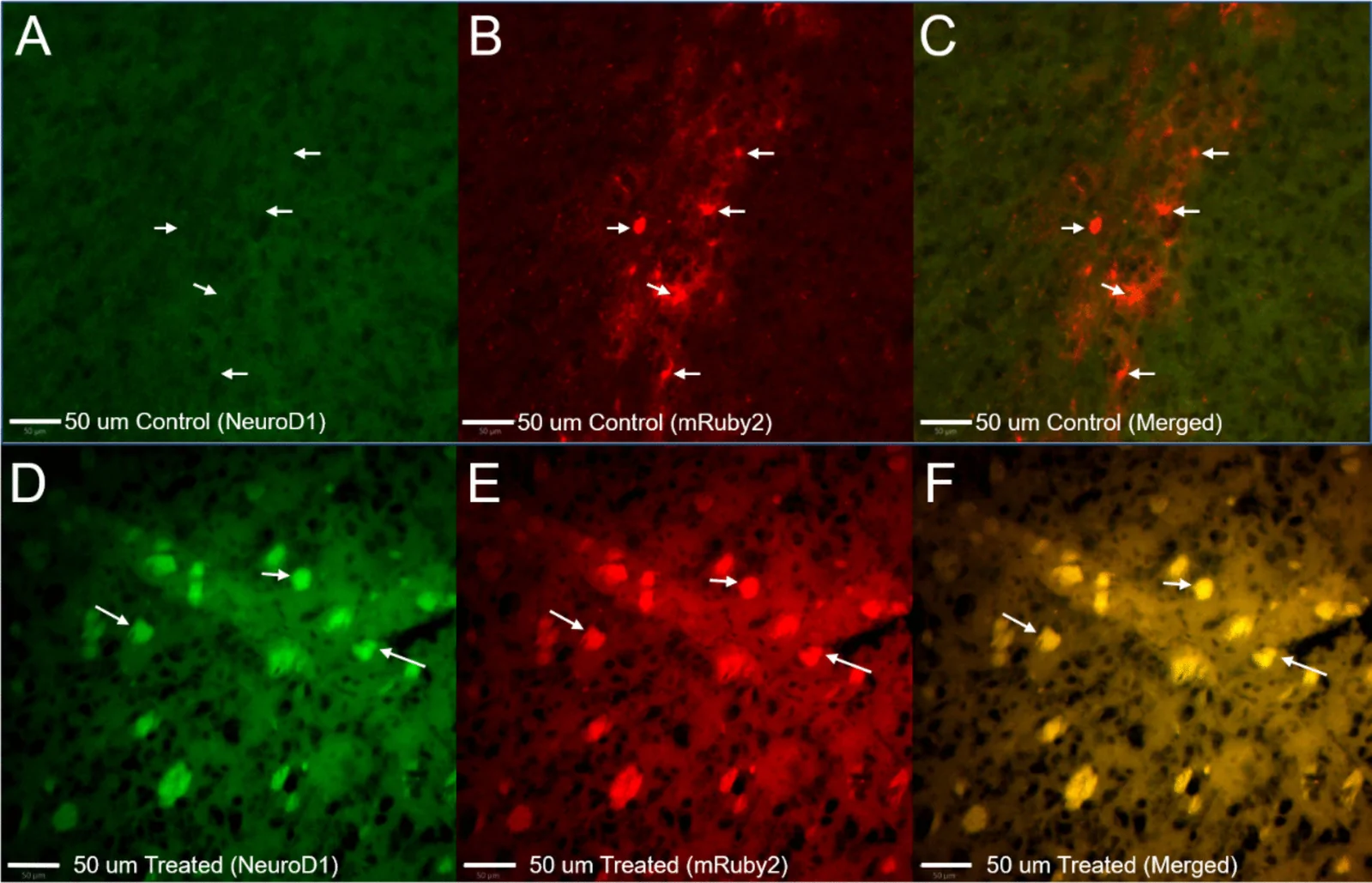
Qualitative depiction of NeuroD1 and mRuby2 co-labeling. White arrows label mRuby2-positive cells. **A** Control tissue with NeuroD1 channel displayed. **B** Control tissue with mRuby2 channel displayed. **C** Control tissue with NeuroD1 and mRuby2 channels displayed. White arrows in **A**–**C** show mRuby2-positive cells that are NOT colabeled with NeuroD1. **D** Treated tissue with NeuroD1 channel displayed. **E** Treated tissue with mRuby2 channel displayed. **F** Treated tissue with NeuroD1 and mRuby2 channels displayed. White arrows in **A**–**C** show mRuby2-positive cells that are colabeled with NeuroD1

**Fig. 9:**
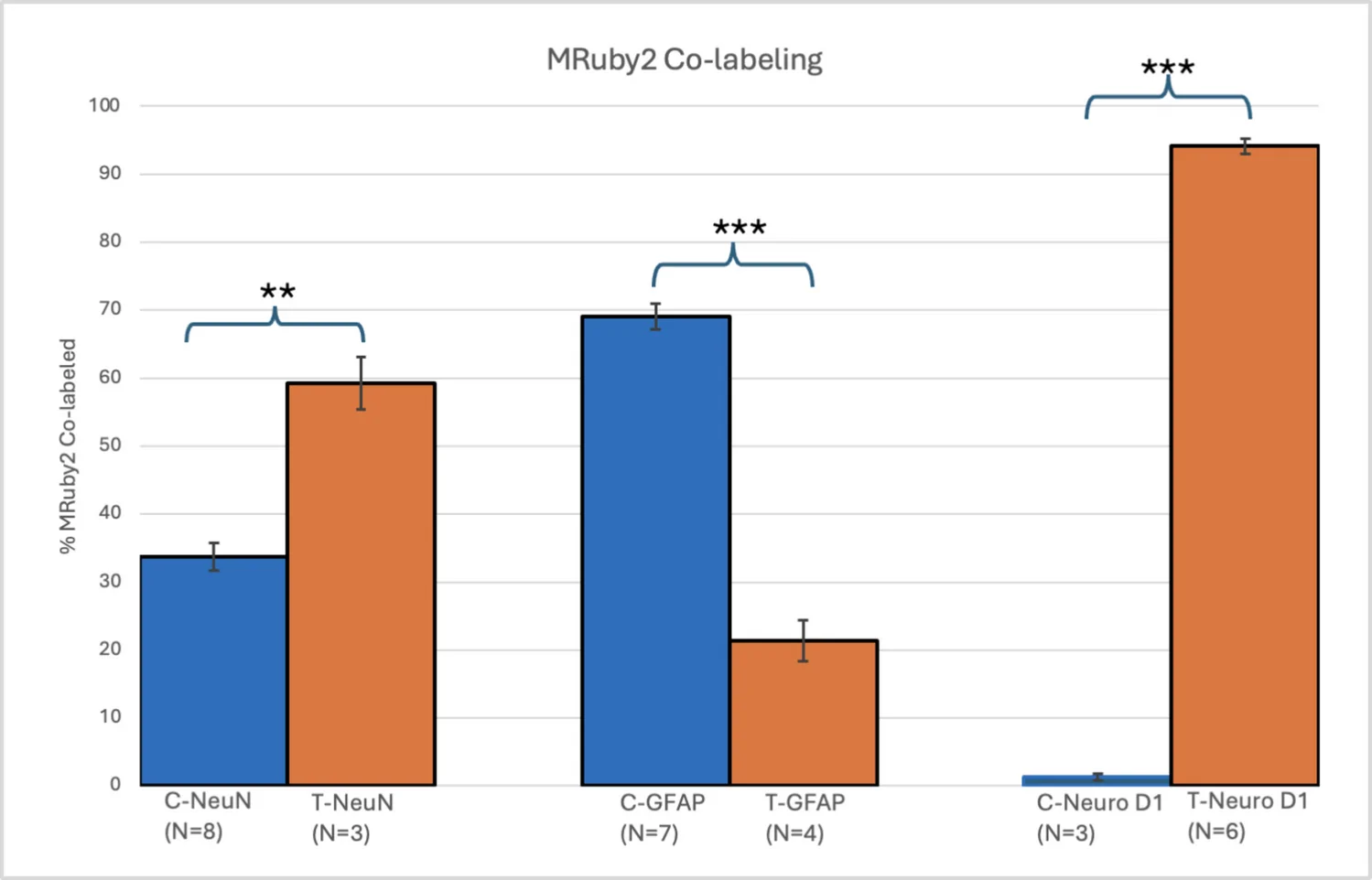
mRuby2 colocalization with NeuN, GFAP, and NeuroD1 in control virus (C) and treatment virus (T) groups at the low dose. Bars represent the percentage of mRuby2-positive cells colabeled with each immunohistochemical marker. NeuN: *p* = 0.0086; GFAP: *p* = 0.000081; NeuroD1: *p* = 1.2 × 10⁻⁸. Not all animals were included in the analysis as acceptable immunohistochemical staining quality was not achieved for every tissue sample. Samples with insufficient staining quality were excluded from quantification

The tissue was stained for NeuroD1 to further demonstrate that the virus was functioning as expected. It was found that mRuby2-positive cells in control samples did not have any NeuroD1 co-labeling present (Fig. 8A–F). This was expected and shows that cells transduced with the control virus did not produce any NeuroD1. In contrast, it was found that, in treated samples, many mRuby2-positive cells also had clear NeuroD1 co-labeling. This confirmed that the treatment virus successfully promoted expression of NeuroD1. Quantification of these results revealed the difference to be statistically significant (Fig. 9).

#### Glial Cell Response

**Fig. 10:**
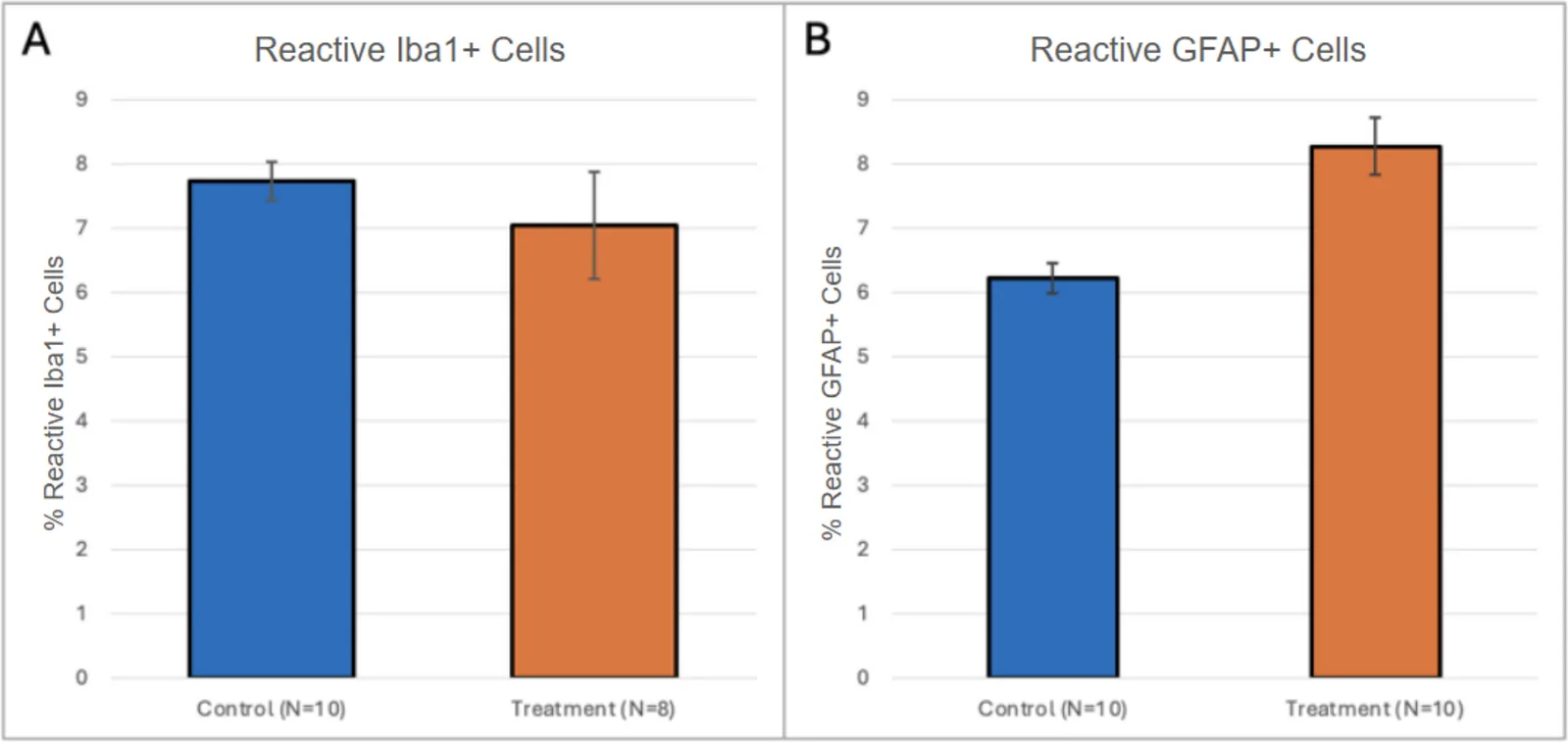
Iba1 or GFAP-positive cells that surpass the morphological reactivity threshold. **A** Percentage of Iba1-positive cells above the morphological reactivity threshold in samples given control virus or treatment virus at low dose (no significant differences). **B** Percentage of GFAP-positive cells above the morphological reactivity threshold in samples given control virus or treatment virus at low dose (no significant differences)

To assess glial responses following TBI, whole coronal slice microscopy tile scans were acquired near the TBI site and stained for Iba1 and GFAP. Quantitative analysis of GFAP-positive and Iba1-positive cell populations was not performed; however, qualitative visual analysis showed no apparent differences between cell populations. Morphological changes in glial cells, astrocytes, and microglia, such as soma and process hypertrophy, have been widely associated with reactive gliosis [14]. Identifying differences in these measures via soma area and perimeter provides a way to assess these altered morphological differences. To identify cells exhibiting morphological features consistent with glial reactivity, a size threshold for cell soma area was established using glial cells located in regions distal to the injury site that lacked visible hypertrophy. These cells were used as a reference population representing baseline morphology. The threshold for morphological reactivity was defined as two standard deviations above the mean soma area of the reference population (threshold = average + 2 × standard deviation). Cells exceeding the threshold were classified as morphologically reactive. This method was based solely on morphological characteristics and should not be interpreted as defining a specific inflammatory phenotype or molecular activation state. This method was also used previously in our lab and found to be an effective means of differentiation [15]. Quantitative analysis of treated and control samples revealed no statistically significant differences, suggesting that NeuroD1 treatment did not measurably alter morphological changes associated with reactive gliosis under the conditions tested (Fig. 10).

**Fig. 11:**
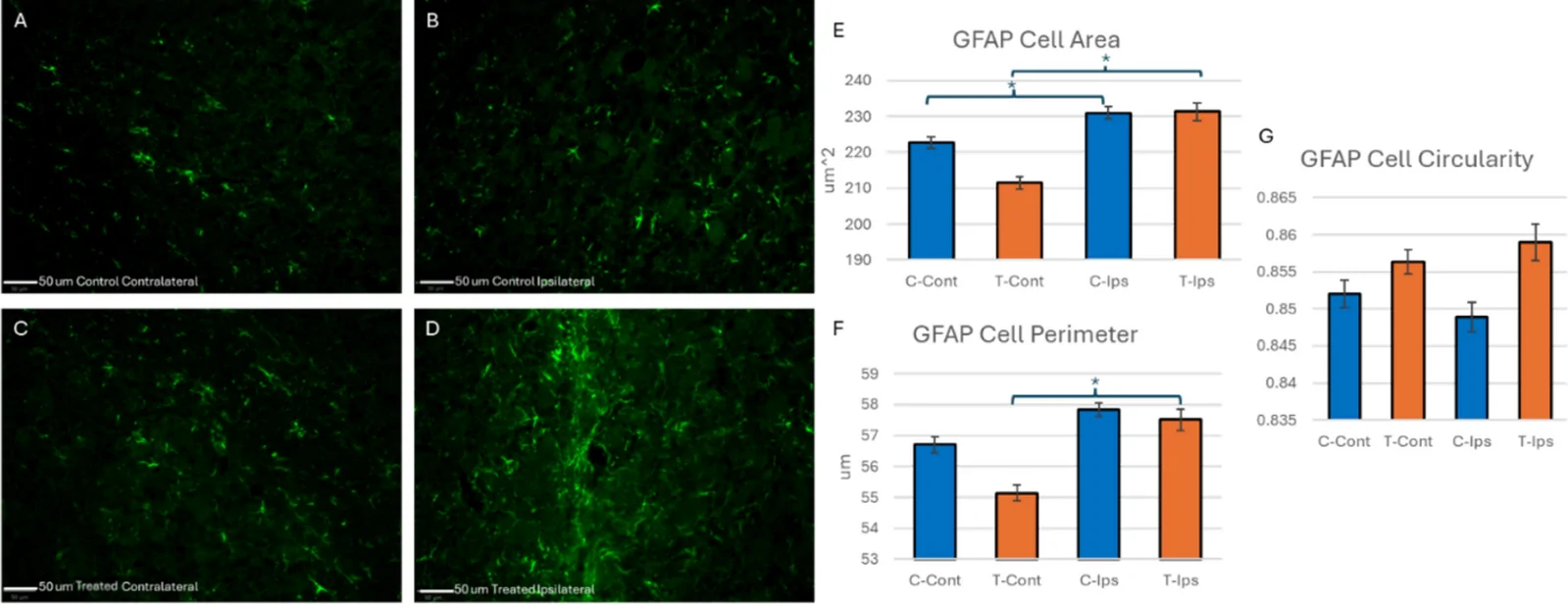
Astrocyte morphology analysis following TBI and treatment. Representative GFAP-stained images showing astrocyte morphology across experimental groups. **A** Control contralateral. **B** Control ipsilateral. **C** Treated contralateral. **D** Treated ipsilateral. **E** Quantification of astrocyte cell area, *p* value for c-cont/c-ips = 0.01491072, *p* value for t-cont/t-ips = 0.028959. **F** Cell perimeter quantification, *p* value for t-cont/t-ips = 0.04876047. **G** Circularity measured, no significant differences noted. Scale bar = 50 µm

We also measured the area and perimeter of glial cells on the TBI ipsilateral side and the corresponding contralateral side to further assess morphological changes. We found that the GFAP-positive astrocyte areas between the contralateral and ipsilateral sides of both control and treated samples had a statistically significant increase. Similarly, the perimeter metrics show a statistically significant increase between the contralateral and ipsilateral sides indicating an increase in size and/or additional/longer processes near the lesion side (Fig. 11A–F). Cell circularity was quantified from the area and perimeter values to further distinguish between resting and morphologically reactive states; however, no significant differences were noted between any groups (Fig. 11G). These findings suggest that the increase in perimeter is representative of a general size increase (hypertrophy), rather than additional/longer processes. Images show the morphological changes in astrocytes, with cells near the ipsilateral side appearing larger.

**Fig. 12:**
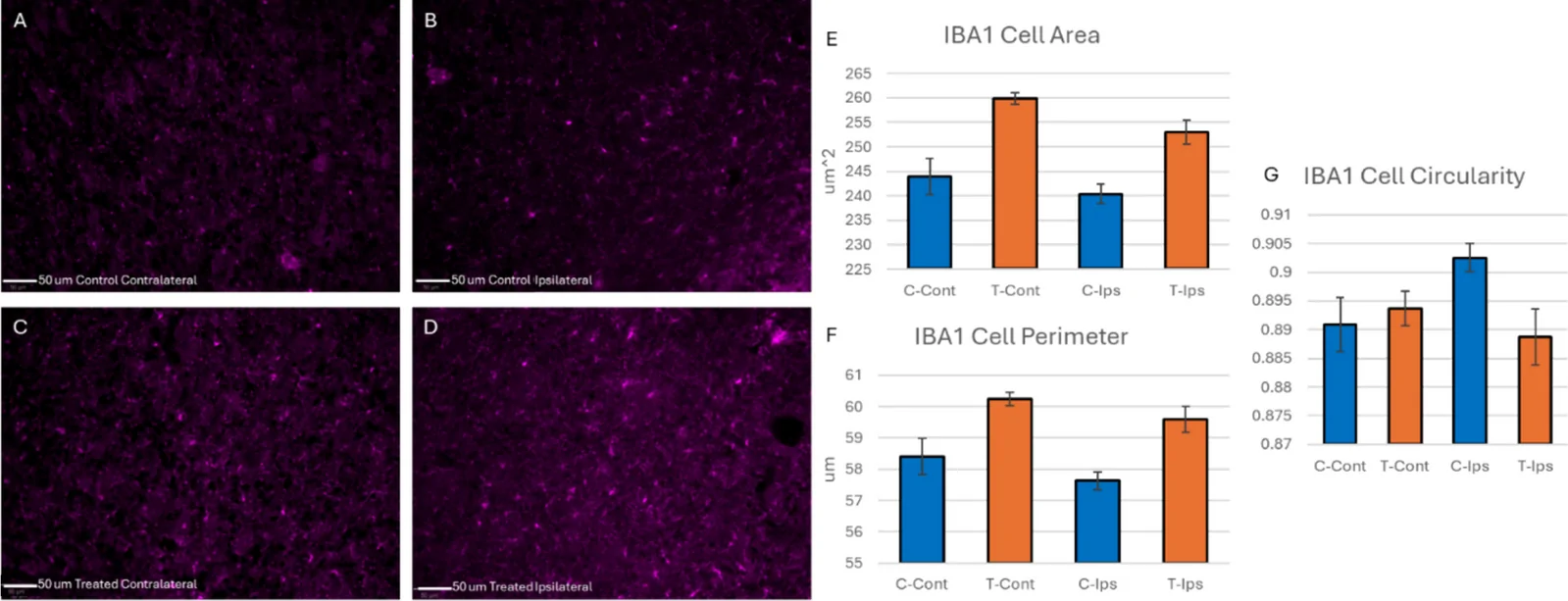
Microglial morphology analysis following TBI and treatment. Representative Iba1-stained images showing microglia morphology across experimental groups. **A** Control contralateral. **B** Control ipsilateral. **C** Treated contralateral. **D** Treated ipsilateral. **E** Quantification of astrocyte cell area, no significant differences noted. **F** Cell perimeter quantification, no significant differences noted. **G** Circularity measured, no significant differences noted. Scale bar = 50 µm

Analysis of Iba1-positive microglia did not show any significant differences in any morphological metrics. This indicates that the treatment had no effect on microglia reactivity. Representative images of the Iba1 tissues show no significant differences (Fig. 12A–G).

#### TBI Lesion Volume and Ventricle Volume with Low Dose

**Fig. 13:**
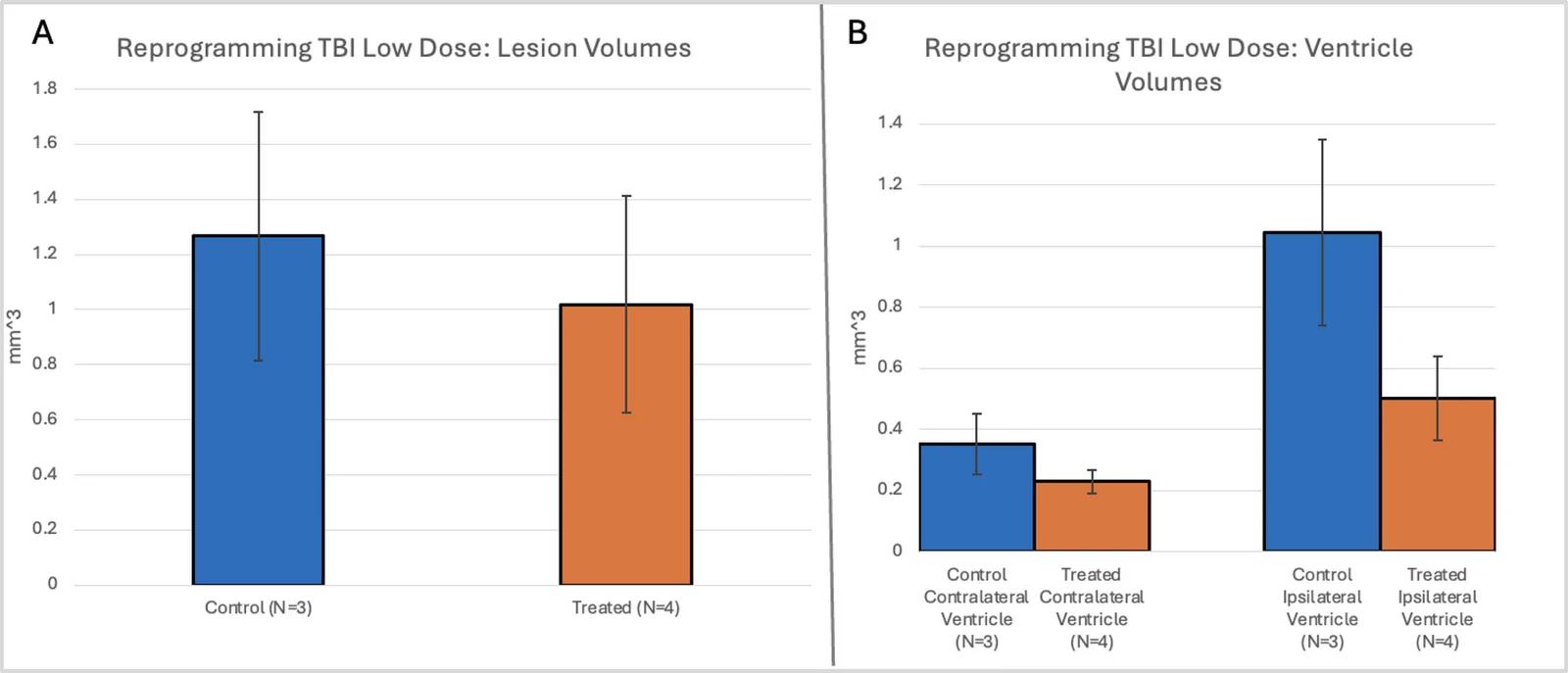
Anatomical analysis of control and treated groups. **A** TBI lesion volumes. No statistically significant differences between treated and control. **B** Ventricle volumes. No statistically significant differences between treated and control

TBI lesion volumes and ventricle volumes were quantified with QuPath annotations. The samples showed no statistically significant differences between the control group and the treated groups in terms of TBI lesion volume and ventricle volume (Fig. 13A, B). This was in agreement with our preliminary results, suggesting that the treatment is unable to provide structural benefits to the brain post-TBI. There is however a general trend of increased ventricle size on the ipsilateral side when compared to contralateral. This is expected, as there should be more tissue atrophy on the injured side than non.

#### Behavior

**Fig. 14:**
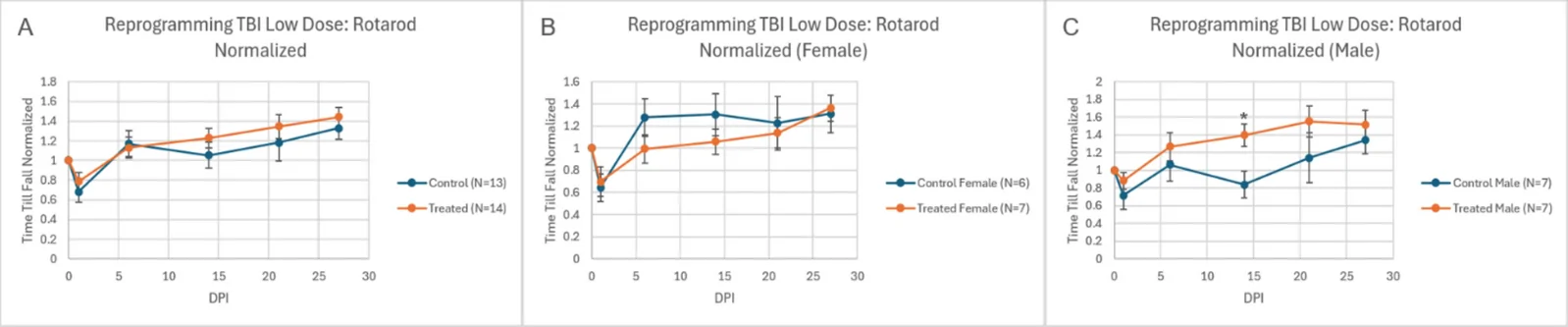
Rotarod results. **A** Normalized rotarod data. No statistically significant differences. **B** Female only normalized rotarod data. No statistically significant differences. **C** Male only normalized rotarod data. DPI 14 *p* value = 0.0114

Neurological data were collected throughout the experiment in the form of rotarod and beam walk tasks. When comparing the rotarod in control females to treatment females, there was no significant statistical difference. However, the treated male group trended better and stayed on top of the rotarod for a longer period of time, noted in 14 days post injury (DPI) (Fig. 14). This improvement is not maintained though, and at the final recorded data point, there are no statistically significant differences between groups. The statistical comparison between male and female groups was not performed in this analysis.

**Fig. 15:**
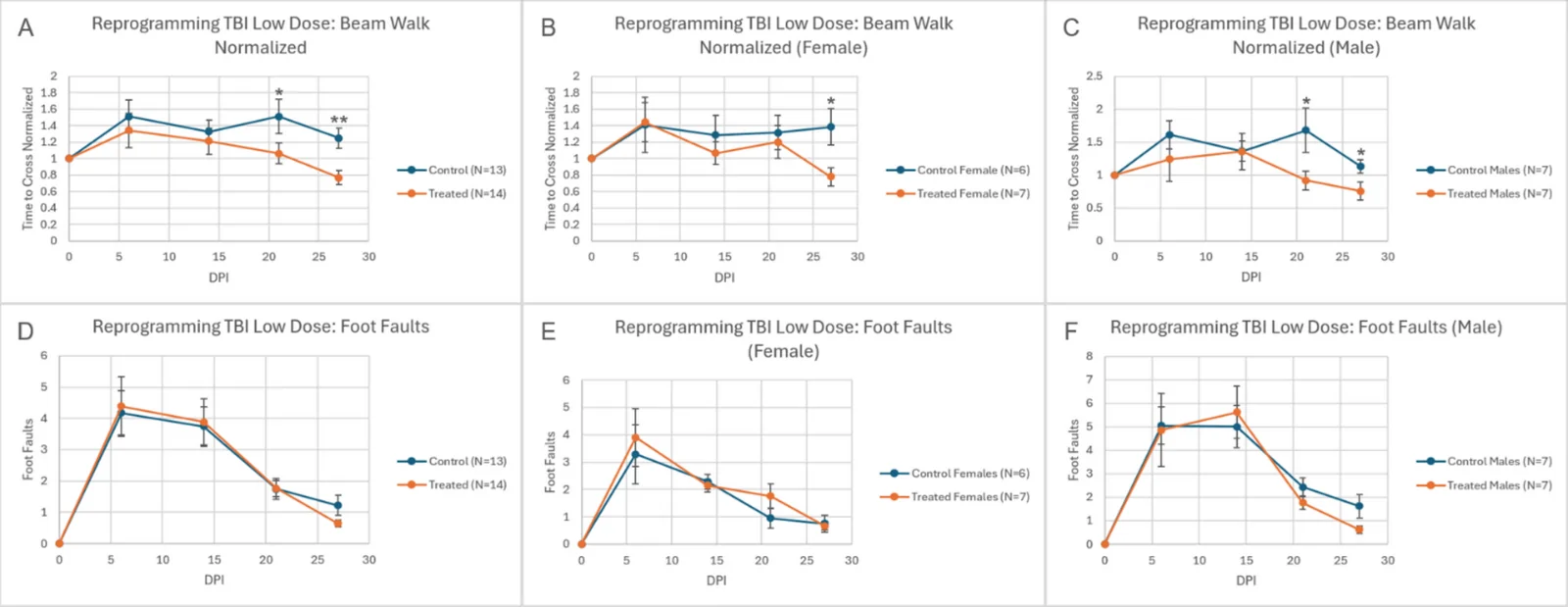
Beam walk results. **A** Normalized beam walk, DPI 21 *p* value = 0.046, DPI 27 *p* value = 0.0026. **B** Female only normalized beam walk, DPI 27 *p* value = 0.0283. **C** Male only normalized beam walk, DPI 21 *p* value = 0.0438, DPI 27 *p* value = 0.0330. **D** Foot fault. **E** Female only foot fault. **F** Male only foot fault

When comparing treatment to control groups in normalized time to cross-beam walk, treated animals had a statistically significant improvement over control at 21 and 27 DPI (Fig. 15). Comparing control females to treatment females in the beam walk, there was a significant decrease in the time treatment mice took to cross the beam walk at 27 DPI. Comparing treated males to control males in the beam walk, treated animals had a significant increase in performance at 6, 21, and 27 DPI. Comparison of the different groups in foot faults revealed no statistically significant differences in performance.

## Discussion

### Alternate Dose Transduction

Conforming with established literature, when exploring the effects of varying the dose of our virus (AAV9-GFAP(short)-Cre and AAV9-CAG-DIO-mRuby2) from 10^11^ to 10^13^ GC/mL, our findings revealed an ideal tendency in which lower doses expressed greater co-labeling of their target astrocyte types. Statistically significant findings were observed that increases in dose had a positive correlation with NeuN-positive colabeled cells and a negative correlation with the number of GFAP-positive colabeled cells. This indicates that to optimize cellular reprogramming of astrocytes into neurons, lower doses such as 10^11^ GC/mL should be used. This should be further explored to determine the mechanisms dictating apparent cell transduction and to what degree higher doses are resulting in toxicity that may be killing cells, as suggested by prior literature [13, 16].

### Astrocyte to Neuron Reprogramming with Low Dose in TBI Models

Given the results of our prior studies, it becomes clear that parameters such as dose are crucial to consider when studying these NeuroD1-based treatments. In our TBI study, we used the information derived from the dosage experiment to design a more optimal experiment in which a low-dose delivery of AAV9-GFAP(short)-Cre and AAV9-CAG-DIO-NeuroD1-T2A-mRuby2 was given for the promotion of astrocyte-to-neuron reprogramming in TBI models. Qualitative and quantitative analysis of the transduced cells in treated animals demonstrated increased NeuN/mRuby2 and NeuroD1/mRuby2 co-labeling with decreased GFAP/mRuby2 co-labeling, while transduced cells in control animals demonstrated increased GFAP/mRuby2 co-labeling, decreased NeuN/mRuby2 co-labeling, and nearly zero NeuroD1/mRuby2 co-labeling. These findings are consistent with NeuroD1-mediated astrocyte-to-neuron conversion. Given the timeline of this study, it is possible that newly reprogrammed neuronal cells did not yet reach a stage of maturity able express detectible levels of NeuN, thereby underestimating the true conversion rates.

No significant differences in astrocyte or microglial morphological changes associated with reactive gliosis were observed between treated and control groups, suggesting similar inflammatory responses. Considering that the reprogramming process is expected to convert transduced astrocytes into neurons, this finding implies that proliferation of glial cells was sufficient to replenish those lost to conversion, thereby preserving morphological ratios comparable to controls. This is in agreement with existing research that suggests glial cell levels are maintained despite cell reprogramming [5]. While the morphology of these astrocytes and microglia cells also had no differences between groups, they did have differences between sides, with ipsilateral cells often having greater size and perimeter. The difference between ipsilateral and contralateral is expected as cells closer to the injury were more likely to become reactive. The reactive gliosis process can be initiated by damage-associated molecular patterns, or DAMPs, that get released when cells are damaged, for example, by a TBI. The signals cause glial cells to begin clearing cellular debris by phagocytosis, which results in the glial cells becoming enlarged and more complex [17]. The higher level of reactive glial cells would be expected in the areas with a higher concentration of DAMPs, which, in this experiment, would likely be the ipsilateral side due to its closer proximity to the impact of the TBI.

No differences in anatomical recovery were observed between the control and treated groups, indicating that the treatment had no structurally restorative effect on the brain tissue. This may be due to the treatment being insufficient on its own to facilitate repair of extracellular structures or the reprogrammed neurons not fully integrating with the affected brain tissue. To fully restore both the functional and structural recovery, additional therapies like growth factors or neuroprotective agents may need to be explored.

No statistically significant differences were observed between the combined male and female treated and control groups during the rotarod task. These findings suggest that, under the conditions tested, NeuroD1 treatment did not produce a consistent treatment-related effect on the motor and sensorimotor functions assessed by the rotarod. However, treated males demonstrated significantly improved rotarod performance compared with control males at 14 days post-injury (DPI 14). The improvement is noted only at DPI 14 and not maintained by the end of the experiment. This implies that the sex-specific improvement does not have a lasting effect on the overall recovery. The sex-specific observation does raise the possibility that treatment responsiveness may differ between males and females following TBI. However, this finding alone does not establish a sex-dependent treatment effect. Future studies specifically powered to evaluate sex as a biological variable will be necessary to determine whether sex influences the response to NeuroD1 treatment.

Interestingly, nearly all animals performed above their preinjury baseline on the rotarod after DPI 6. This finding may reflect insufficient pre-experimental training or continued motor learning during the study. Future studies should therefore consider extending preinjury rotarod training, potentially to a duration comparable to the experimental period, to establish a more stable performance baseline and minimize the influence of learning effects.

Significant improvements in beam walk performance were observed in both male and female animals, suggesting that NeuroD1 treatment may facilitate functional recovery following TBI. Notably, these functional improvements were observed despite the absence of clear gross anatomical restoration in treated brains. One possible explanation is that NeuroD1 treatment may promote neuroplasticity and functional circuit remodeling that are not readily reflected by gross anatomical changes. Previous studies have demonstrated that NeuroD1 can alter the post-injury cellular microenvironment, including through increased production of neurotrophic factors such as pleiotrophin [18]. Pleiotrophin has been reported to exert neuroprotective effects and promote axonal outgrowth following neuronal injury [19], suggesting that NeuroD1-mediated modulation of neurotrophic signaling may support axonal remodeling and functional recovery after TBI. Although the present study did not directly assess circuit remodeling or synaptic integration, these mechanisms may contribute to the functional improvements observed following NeuroD1 treatment.

Several limitations should be considered when interpreting the findings of this study. First, glial reactivity was assessed using morphological criteria rather than molecular markers associated with specific activation states or inflammatory phenotypes. Although changes in soma size and cellular morphology are commonly used indicators of astrocyte and microglial responses following injury, these measures cannot distinguish between distinct functional or inflammatory states. Additionally, lineage tracing was not performed to definitively determine the cellular origin of NeuroD1-associated neuronal cells. Consequently, the extent to which the observed neuronal labeling reflects direct astrocyte-to-neuron conversion or other cellular mechanisms cannot be definitively established. Furthermore, the maturation, functional activity, and synaptic connectivity of the reprogrammed neuronal cells were not assessed. Therefore, it remains unclear whether these cells functionally integrate into existing neural circuits or contribute directly to the behavioral improvements observed in this study. Finally, the observation that many animals performed above their preinjury rotarod baseline suggests that motor learning during the experimental period may have influenced rotarod performance, highlighting the importance of more extensive preinjury training or a predefined performance plateau in future studies.

Future research should focus on optimizing experimental parameters, incorporating additional behavioral and molecular analyses to better understand the precise neurological benefits and the mechanisms prompting them, and evaluation of supposedly reprogrammed cells to assess their viability and functionality. Incorporation of markers for reactive astrocyte phenotypes, inflammatory microglial phenotypes, and alternative subtypes would provide a more comprehensive characterization of glial responses to NeuroD1 treatment. Markers for developing neurons such as doublecortin or polysialylated neural cell adhesion molecules could provide additional insights toward maturation levels and true conversion rates. Assessments of neuronal viability and integration could be performed via synaptic markers such as MAP2 and synaptophysin with confocal microscopy, while whole cell patch clamp tests could be used to assess functionality. Additionally, exploring combinatorial approaches, such as pairing delivery of NeuroD1 for cellular reprogramming with neuroprotection and/or neuromodulation approaches, may provide even greater benefits.

## Conclusion

The results of this study show that AAV-mediated delivery of NeuroD1 intended for reprogramming can provide neurological benefits when used to treat TBI. To ensure consistent research results, several factors, including virus dose, the timing of administration, and the specific injury model used, must be considered and optimized.

While NeuroD1 treatment did not significantly alter neuroinflammatory measures or gross anatomical recovery of the brain, it did result in neurological improvements in beam walk task performance. This suggests that this treatment may enhance functional recovery by promoting neural plasticity rather than directly preserving or restoring brain architecture. Our research also validated and supported the prior literature in the field, showing that lower virus doses result in improved transduction specificity. Further investigation should be conducted to determine if increases in specificity result in improved rates of cellular reprogramming and what the underlying mechanisms are for improved neurological performance.

Our research provides a promising foundation for the continuation of NeuroD1-based treatment approaches in TBI models. Future studies should assess the long-term stability, integration, and functional contribution of reprogrammed neurons. These future studies could be facilitated by long period behavioral tests and electrophysiological recording, like patch-clamp techniques [11]. Lastly, developing clinically relevant delivery methods, such as intravenous injection rather than cranial, and testing in larger animal models are crucial next steps in translational research.

## Data Availability

Data Available Upon Request.
